# Identifying Current Practices and Areas for Improvement in Medication Management During Care Transition Through an Interprofessional Collaboration Framework

**DOI:** 10.2147/JMDH.S528819

**Published:** 2025-07-30

**Authors:** Léa Solh Dost, Gaëlle Maillard, Evelina Cardoso, Marie P Schneider

**Affiliations:** 1 School of Pharmaceutical Sciences, University of Geneva, Genève, Switzerland; 2 Institute of Pharmaceutical Sciences of Western Switzerland, University of Geneva, Genève, Switzerland; 3 Pharma24, Academic Community Pharmacy, Geneva, 1205, Switzerland

**Keywords:** interprofessional collaboration, healthcare professionals, outpatient care, continuity of patient care, medication therapy management

## Abstract

**Purpose:**

Poor coordination and communication during care transitions can lead to medical errors, patient dissatisfaction, and hospital readmissions. The transition period from hospital to the first medical appointment is a high-risk and vulnerable time for patients, and a complex one for healthcare professionals. While interprofessional collaboration can improve the quality and safety of care, its implementation remains underexplored. This study examines the current state and areas for improvement in interprofessional medication management during the hospital discharge transition (from hospital discharge to first medical appointment) for patients self-managing their medications.

**Methods:**

A qualitative study was conducted using a serial focus group methodology with patients and healthcare professionals from hospital and community settings. Participants were sampled purposively. Discussions were audio-recorded, transcribed verbatim, and analysed using inductive thematic analysis. Thematic findings were categorised using the 2010 Canadian National Interprofessional Competency (CIHC) Framework, distinguishing between current practices and areas for improvement. Additionally, a classification questionnaire, adapted from the nominal group technique, was used to rank proposed improvement strategies based on their perceived impact and feasibility.

**Results:**

Twelve participants (10 healthcare professionals and two patients) contributed to four focus groups. The study identified strengths and areas for improvement in five of the six CIHC 2010 competency domains: 1. Interprofessional communication: present but needing better structure and proactivity; 2. Patient partnership: recognised but requiring more consistency; 3. Role clarification: unclearly defined, causing inefficiencies; 4. Team functioning: common in hospital settings, but inconsistent during transition; 5. Collaborative leadership: present but lacking clear coordination at handover. An overarching category, “Macro-level improvements” was introduced to highlight system-wide changes and the need for policy support to implement and sustain interprofessional collaboration.

**Conclusion:**

While existing practices emphasise interprofessional communication and patient involvement, role clarity and collaborative leadership remain significant challenges. Healthcare professionals are motivated and ready to collaborate, but policy and coordinated efforts among healthcare meso- and macro-entities are needed to implement sustainable interprofessional practice models, to increase quality of pharmaceutical care, and improve patient outcomes during care transition from hospital to home.

## Introduction

Transitioning from hospital to outpatient care is a critical and high-risk phase in the healthcare journey of patients with multiple long-term diseases and polypharmacy.^1–3^ Poor coordination and communication among healthcare professionals during care transitions can lead to medical errors, patient dissatisfaction, re-hospitalization, and increased healthcare costs.^2^ Effective care transitions require comprehensive interprofessional collaboration, efficient communication between all healthcare professionals and patient education to ensure continuity of care.^4^,^5^ Despite these known challenges, implementing best practices in terms of collaborative practice remains insufficiently addressed in many healthcare settings.^6–8^

Medication management involves partnering with the patient to optimise safe, effective and appropriate drug therapy.^9^ Medication management is complex during hospitalisation and post-discharge, as patients’ medication regimens often undergo significant changes during care transition.^10^,^11^ Patients may be faced with multiple drug-related problems, such as unintentional medication discrepancies, drug interactions, lack of understanding or poor adherence, jeopardising medication safety and effectiveness.^12–15^ Furthermore, the accurate transfer of medication information across healthcare settings remains challenging, increasing the risk of discontinuity of care.^16^,^17^ While health information technology, such as electronic patient records, can secure care transition, several barriers, such as data security and patients’ reluctance, hinder its adoption in countries such as Switzerland.^18–20^

Interprofessional collaboration or collaborative practice happens when multiple health workers from different professional backgrounds work with patients, families, carers, and communities to deliver the highest quality of care across settings.^21^

Interprofessional collaboration is a promising strategy to enhance the quality and safety of care transitions.^22–25^ However, various factors can hinder interprofessional collaboration, such as lack of standardised protocols, poor communication, lack of awareness of each other’s roles, and previous work experiences in silos.^13^,^26–29^ Several frameworks describe and classify key competencies for interprofessional collaboration.^30^,^31^ Among these, the Canadian National Interprofessional Health Collaborative (CIHC) Framework has been widely adopted in academic settings and interprofessional education, as it emphasises transversal, team-based competencies and serves as a foundation for competency development across professions.^32^ The 2010 CIHC Framework organises interprofessional collaboration into six competency domains: interprofessional communication, patient/client/family/community-centred care, role clarification, team functioning, collaborative leadership and interprofessional conflict resolution.

While qualitative methodologies have been employed to explore the care transition process from hospital to outpatient care,^14^,^33–36^ a notable gap remains in the literature regarding interprofessional collaboration across inpatient and outpatient settings.^29^ The existing literature focuses mainly on the perspectives of healthcare professionals within a single care setting or targets specific groups of healthcare professionals.^29^ However, the complexity of interactions between various healthcare settings and the factors influencing collaboration during transition of care remain insufficiently understood.^37^,^38^ Previous studies highlight that, while multiple healthcare professionals are involved in care transition, their efforts are often poorly coordinated, placing a heavy burden on patients when they return home after hospital discharge without professional support for medication management (eg home nurses).^7^,^37^ These findings raise questions about the specific roles of healthcare professionals in the transition of patients from hospital to their first medical appointment, prompting us to explore interprofessional practices and perspectives on medication management during this transition Therefore, this study examines the current state and areas for improvement in interprofessional medication management during the hospital discharge transition (from hospital discharge to first medical appointment) for patients self-managing their medications.

## Methods

This study followed the Consolidated Criteria for Reporting Qualitative Research (COREQ).^39^ The study was approved by the University Commission for Ethical Research in Geneva (CUREG-MM-2022-05-91). This study was conducted in accordance with the ethical principles of the Declaration of Helsinki.

### Study Design

This qualitative study consisted of serial focus groups with healthcare professionals and patients from in- and out-patient settings. The focus group methodology was chosen to facilitate discussion and the exchange of perspectives among participants.^40^ In serial focus groups, the same groups are reconvened multiple times over time, prioritising the depth of discussion.^41^

### Study Setting and Context

This study was conducted in the canton of Geneva (500’000 inhabitants), Switzerland. Geneva’s healthcare landscape is centered around the Geneva University Hospitals (Hôpitaux universitaires de Genève, HUG), one of the largest university hospital groups in Switzerland, comprising eight public hospitals, two clinics, and around forty outpatient care centres.

Switzerland’s healthcare system is characterised by its liberal and decentralized structure. Universal health coverage is guaranteed through different health insurance companies, but each of the 26 cantons exercises significant autonomy in organizing and financing healthcare services.^42^ This results in notable regional variations in care pathways, access to services, and the adoption of health information technologies. A key feature of the Swiss system is the principle of free choice which extends to health information management, where patients must actively opt in to electronic health records (EHRs) and retain complete control over which healthcare professionals can access their health data. This is one cause of low patient and healthcare professional adoption, resulting in less than 2% of the population enrolled in the EHRs as of 2022.^43^

The typical care transition process for patients discharged home without professional medication management involves several key healthcare professionals: hospital physicians, nurses and pharmacists; community pharmacists and their team; and general practitioners (GP). The GP typically receives a discharge letter and summary, but the content and format of these documents vary widely depending on the hospital and the information systems used.^44–46^ The community pharmacist is the first healthcare professional seen at discharge.^7^ They receive discharge prescriptions directly from patients but frequently lack access to essential clinical information, which places them in a crucial yet challenging position for ensuring the continuity and safety of care.^47^ While interprofessional collaboration in outpatient care is still emerging in Switzerland, there is a clear and rising interest in fostering and evaluating these collaborative approaches.^48^

### Population and Recruitment

Healthcare professionals and patients participated in the focus groups. Inclusion criteria for healthcare professionals were their involvement in medication management of polypharmacy patients in the HUG hospital or in the outpatient setting in Geneva, Switzerland. Inclusion criteria for patients were patients living with at least two chronic conditions and having experienced an unplanned hospitalisation.

A purposive sampling methodology was applied. Healthcare professionals of different professional backgrounds, work experiences, and genders were recruited by email. Patients were patient partners identified by the HUG Patients Partners platform, which aims to facilitate the implementation of the partnership between patients, caregivers, and professionals. “Patient partners” are defined as patients with lived experience of a health condition involved in academic-related activities.^49^ All participants were contacted before the focus groups to obtain written consent and answer questions.

### Researcher Characteristics

Focus groups were moderated by GM (Master student in pharmacy) or LS (PhD Student and community pharmacist trained in qualitative research) and co-animated by EC or CB (Post-doctoral fellows trained in qualitative research). Researchers were unknown to participants, except for two participants who knew LS from professional encounters.

### Procedures

**Figure 1:**
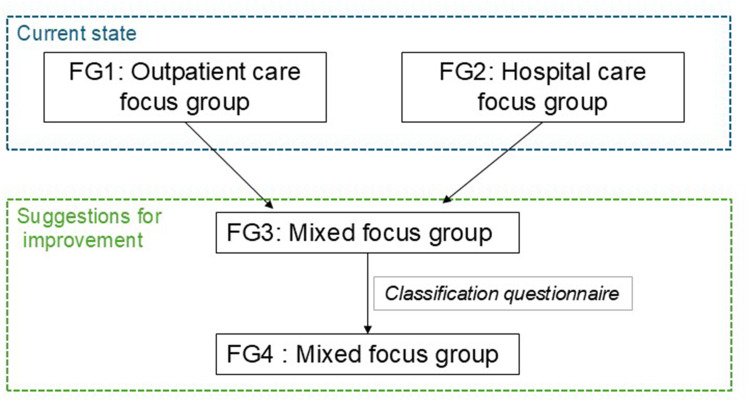
Conduct of focus groups (FG) with patients and healthcare professionals; mixed: hospital and outpatient care focus group.

**Abbreviation**: FG, focus group.

The focus groups (FGs) were conducted as described in Figure 1 in French. FG1 and FG2 were conducted to explore the current state of interprofessional collaboration regarding medication at hospital discharge. FG1 included outpatient healthcare professionals and patients, while FG2 involved hospital healthcare professionals and patients. To encourage open and honest discussion of setting-specific challenges and needs, the initial focus groups were conducted separately by care setting. FG3 and FG4 shifted the discussion towards identifying areas for improvement, each composed of a mix of outpatient and hospital healthcare professionals and a patient. Before FG4, participants received a classification questionnaire, adapted from the nominal group technique.^50^ Each participant assigned a score to the items on a scale from 1 to 11 for both criteria: impact (with 11 representing the most impactful and 1 the least impactful) and ease of implementation in practice (with 11 representing the easiest and 1 the hardest to implement). The individual scores for each item were then summed across all participants to determine the overall ranking of each suggestion.

### Data Collection

FGs were held in person at the University of Geneva, Switzerland. Three semi-structured interview guides, developed for this research and available in Figures S1–S3, helped guide the sessions. They addressed key topics, including current practices and challenges related to interprofessional collaboration in medication management at hospital discharge, the roles and responsibilities of different healthcare professionals, patient involvement, and communication processes between inpatient and outpatient settings. Subsequent focus groups examined areas for improvement, identified resources necessary to enhance collaboration, and gathered participant views on proposed solutions.FGs were audio-recorded on two devices, and field notes were taken to facilitate moderation and transcription. Recordings were transcribed verbatim and manually by GM and checked for accuracy by LS. Data was pseudo-anonymized, and audio recordings were destroyed after analysis. The classification questionnaire was developed using the institutional Limesurvey^®^, an open-source online survey tool.^51^

### Data Analysis

Transcripts were coded using MAXQDA™ 2018, Release 18.2.3. Analysis was conducted in French, and the verbatim information of interest was translated into English and validated by a native English-speaking researcher to preserve the meaning. An inductive thematic analysis, as described by Braun and Clarke,^52^,^53^ was undertaken by the research team to analyse the data. Transcriptions were coded into themes and subthemes in a systematic, comparative, and iterative manner. For each theme, data were categorised according to whether they described current practices or areas for improvement, regardless of the focus group concerned. To ensure the validity of the coding method, all transcripts were double-coded and compared to reach a consensus on the final themes. The analysis was conducted in parallel with focus groups to ensure data saturation. Thematic saturation was considered to have been achieved when no new code or theme emerged and new data repeated information that has already been coded.^54^ Themes were organised into six categories, according to the 2010 CIHC Framework:^32^ 1. Interprofessional communication, 2. Patient partnership and shared leadership, 3. Role Clarification, 4. Team functioning, 5. Collaborative leadership, and 6. Interprofessional conflict resolution. For each category, we reported A. the current state and B. potential options for improvement. Notably, “Patient partnership” was chosen over “patient-centred care” as referenced in the CIHC framework, to reflect the evolving role of patients as active partners in their care.^55^,^56^ As our research was undertaken before the release of the 2024 CIHC update,^57^ we adopted the 2010 CIHC Framework.

## Results

Fifteen participants were contacted directly, and the announcement was forwarded to the hospital physicians’ and nurses’ Email list. Twelve participants (characteristics in Table 1) participated in the four focus groups between September 2022 and March 2023. The mean duration of the focus groups was 92 minutes (SD:13).

**Table 1:** Participants’ Characteristics and Participation in Focus Groups (FG)

Setting	Representative of	Gender	Experience [Years]	Participation in			
				FG1	FG2	FG3	FG4
Outpatient	General practitioner	Female	25	x			
Outpatient	General practitioner	Male	10	x		x	x
Outpatient	Community pharmacist – head pharmacist	Female	25	x		x	x
Outpatient	Community pharmacist – head pharmacist	Female	8	x			
Hospital	Hospital physician – chief resident	Female	8		x		
Hospital	Hospital physician – internist	Female	4				x
Hospital	Nurse – ward nurse	Female	8		x	x	x
Hospital	Nurse – Itinerary patient manager	Male	5		x	x	x
Hospital	Hospital pharmacist – clinical pharmacist	Female	14		x	x	
Hospital	Hospital pharmacist – project leader	Female	22		x	x	x
–	Patient	Female	–	x			x
–	Patient	Female	–		x	x	

**Figure 2:**
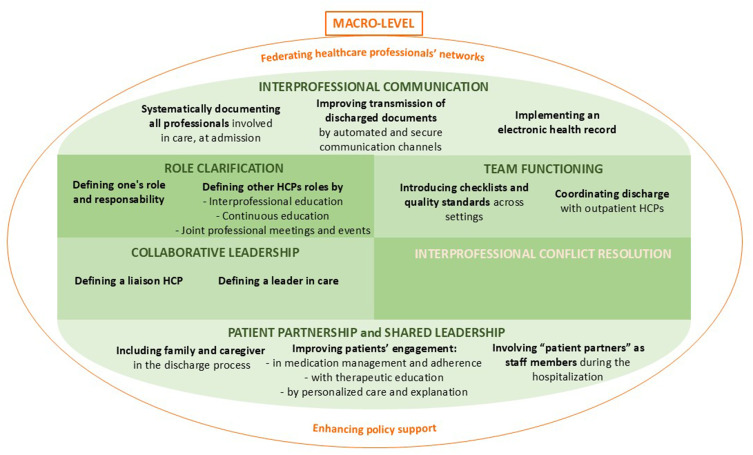
Areas for improvement in interprofessional collaboration around medication management during care transition, classified according to the CIHC Framework.^32^

**Abbreviations**: HCP, healthcare professionals.

Results are structured according to the six competency domains of the CIHC Framework, distinguishing between current practices and potential areas for improvement within each category. Additionally, an overarching category, “Macro-level improvements”, was introduced to capture broader strategies and system-level changes that could enhance all six CIHC competencies. Participants’ rankings of options for improvement are presented in Table 2, along with their impact and ease of implementation. Figure 2 illustrates areas for improvement in interprofessional collaboration around medication management during care transition, classified according to the CIHC Framework.

**Table 2:** Agregated Ranking of Areas for Improving Interprofessional Collaboration According to Impact and Ease of Implementation

Areas for Improving Interprofessional Collaboration During Care Transition	Impact	Ease
Transmitting discharged documents in a timely manner	11	6
Implementing the electronic health record for all patients	10	3
Coordinating patient discharge with outpatient healthcare professionals before discharge	9	8
Improving the quality and standardisation of discharged documents	8	5
Actively involving patients as partners in care	7	4
Using the opportunity of the hospital stay to list all the professionals involved in the patient’s care	6	9
Improving patient education	5	11
Defining a care coordinator in the existing healthcare team	4	7
Defining the roles and skills of all involved healthcare professionals (eg through interprofessional meetings and training)	3	10
Establishing an additional liaison healthcare professional	2	1
Implementing medication and patient monitoring services in outpatient care (eg chatbot after hospital discharge)	1	2

**Notes**: Ranking from 1 to 11: the least to the most impactful; the hardest to the easiest.

### Interprofessional Communication

#### Current State of Interprofessional Communication

Participants reported they communicate reactively when questions or challenges arise, such as gathering extra information, conducting medication reconciliation, scheduling follow-up appointments, or obtaining medication details, typically initiated by phone, email, or fax. They acknowledged that information transfer during the care transition had improved over the years with a particular willingness to improve the quality of transition documents and to share them on time:

“I believe a willingness for interprofessional collaboration is beginning to take shape. Currently, integrating information systems remains complex, but [...] there is a clear intent to share information and communicate effectively, all driven by a common goal: ensuring patient safety.” – Patient 1, FG1

Healthcare professionals are often hard to reach, especially in the hospital setting, and the diversity of stakeholders involved in patients’ care and the rotation of interns are perceived as barriers to effective communication:

We sometimes get calls from community pharmacists, and we’d like to answer them, but [...] the GP doesn’t know, the cardiologist doesn’t know. [The internist] is long gone, so […] it’s complex to redirect the call – Nurse, FG2

A national electronic health record is being introduced, but participants highlighted several challenges in its implementation, as detailed in Table 3. Without a fully functional and connected electronic health record system, information must be manually transcribed multiple times across different platforms. This duplication increases the risk of incomplete medical records, leading to potential errors.

**Table 3:** Difficulties Mentioned by Participants in Implementing a Shared Electronic Patient Record

**Patient level**	Poor communication on the use and benefits of the electronic health record
	Concerns about data security and confidentiality
	Lack of user-friendliness, poor usability
**Healthcare professionals level**	Current use of other local and unconnected healthcare software
	There are no clear guidelines on the use of electronic health records for hospitalised patients.
**System-level**	No obligation to enrol patients
	Free choice for patients to divulge information to all or certain healthcare professionals
	Technical difficulties in connecting and interfacing the different software (lack of interoperability)

#### Improving Interprofessional Communication

Participants suggested that the prescriptions and patients’ biopsychosocial information should automatically be communicated to all healthcare providers, such as GPs, pharmacists, home care nurses, social workers, and the patient. To facilitate interprofessional communication, all healthcare providers should be identified in the patient’s hospital record on admission. To enable efficient and quick communication, a participant suggested setting up a centralised, automated, and secure information transmission channel, such as an instant messaging:

We should have an updated and automatic system […]. If I do a medication intervention, the pharmacist knows about it, and the home-care nurses know about it automatically. *–* GP, FG4

Participants identified several strategies to enhance the implementation and acceptance of the electronic health record. They emphasised the need for improved communication with patients, particularly regarding patient safety and enhanced interprofessional collaboration. A gradual implementation approach was preferred, starting with a shared electronic medication plan and gradually expanding to a comprehensive health record. Others suggested prioritising polypharmacy patients, who are at higher risk for medication errors and hospital readmissions, as early adopters of the system:

I think the people with the most to gain from this record are complex patients. They’re not going to say no to a system that makes life easier for the healthcare professionals around them. – GP, FG3

### Patient Partnership

#### Current State of Patient Partnership

All participants emphasised the importance of engaging patients as active partners in their care, viewing this collaboration as crucial for accurately assessing patient needs, delivering patient-centred care, and ensuring safety during care transitions. However, they also identified several challenges, including patients’ varying willingness to engage in care and the limited time and resources available to healthcare professionals. Participants distinguished between two types of patients: those who are proactive and actively involved in their care and those who are more passive and prefer not to take an active role:

[As a patient,] I want to understand why. My doctor always gives me some explanations, […] and I ask questions […], but I know some patients aren’t interested. – Patient, FG1

Patient education and empowerment were mentioned as tools to engage patients in their care actively:

It’s important to start with the patient’s needs, and understand them, then build with the patient, so that the patient can acquire more skills, get involved, and make decisions. – Hospital pharmacist, FG2

The value of collaboration was further illustrated by a patient who described how openness from healthcare professionals influences their sense of reassurance and trust:

If the healthcare professional is open to us finding a solution together, that reassures me. But I’ve been through periods where doctors would say to me, ‘No, it’s this and nothing else,’ and that’s terrible, you lose trust – Patient, FG2

Participants noted that they frequently involve family caregivers in the care process, particularly for elderly patients or those unable to manage their medications. They highlighted caregivers as a valuable resource in both inpatient and outpatient settings, providing essential information about medication history and any changes in medication.

#### Improving Patient Partnership

Participants agreed that patients should be more empowered and actively integrated into the healthcare team by increasing their involvement in medication understanding and management through targeted therapeutic education programs and patient empowerment campaigns. Additionally, medication explanations should be more personalised to meet individual patient needs. Some participants suggested better inclusion of family caregivers, particularly for high-risk patients, to support care further. One patient emphasised the importance of healthcare professionals adapting their communication style to suit each patient and continuously improving their communication skills to foster better understanding and engagement:

Yes, empowering the patient is great, but it also depends on how [the HCP] gives the information. I think there’s room for improvement in communication skills. [of healthcare professionals] – Patient, FG4

One patient suggested enhancing patient engagement by involving “patient partners” as hospital staff, who could meet with patients during their hospitalisation to better vulgarise information. Upon discharge, some participants suggested equipping patients with a chatbot-based application to allow patients to ask questions about care or medications and refer patients, if needed, to a teleconsultation.

### Role Clarification

#### Current State of Role Clarification

All participants acknowledged having limited knowledge about the roles and responsibilities of other healthcare professionals during care transitions. While they recognised that each healthcare professional brings unique expertise in medication management, they felt that these complementary strengths are not fully utilised in practice and are not effectively coordinated:

The doctor will identify prescription errors; the pharmacist will identify interactions or errors in the regimens. If we were all looking at the same document, we could all contribute with our expertise [...] but for now, it’s fragmented. – Community pharmacist, FG1

#### Improving Role Clarification

To improve care coordination during and after discharge, healthcare professionals’ roles, skills, and responsibilities must be more clearly defined and collaboratively integrated within the healthcare ecosystem. Participants emphasised that this process begins with each healthcare professional clearly understanding their role within the team:

First, we must define who we are and then learn more about each other. […] It’s important to be aware of everyone’s expertise and roles, and for example, when you ask for help […], you can say: «That’s where my competencies end, and here is where yours begin» – Hospital pharmacist, FG2

Understanding the roles and responsibilities of the different healthcare professionals could be improved by interprofessional education during academic and continuous training, joint professional meetings, or events.

### Team Functioning

#### Current State of Team Functioning

In the outpatient setting, interprofessional coordination and collaborative work were mentioned less frequently than in the hospital setting, where participants emphasised the close teamwork among the itinerary patient manager, nurses, and physicians. Discussions highlighted specific coordination practices that could be implemented to enhance continuity between hospital and outpatient care, such as agreements between hospital nurses and pharmacies to maintain a stock of essential medications, ensuring seamless transitions at discharge.

Participants also observed that structured collaborative practices were more established for patients receiving care from home-care nurses than those managing their medications autonomously. Raising awareness about the importance of teamwork and strengthening collaborative practices helped participants improve coordination and interprofessional cooperation. However, significant barriers to seamless teamwork between inpatient and outpatient settings persist, particularly due to limited bridging resources such as time and staffing constraints in both environments.

Participants noted that these two settings often function in silos, with minimal coordination between them. Additionally, the degree of involvement in ensuring continuity of care varied among healthcare professionals, influenced by factors such as individual motivation, pre- and post-graduate training, and the organisational structure of their workplace:

Not all wards have an Itinerary Patient Manager or similar resources. Not all doctors and nurses are necessarily […] well aware of the difficulties of medications reconciliation and continuity of care. – Hospital pharmacist, FG1

#### Improving Team Functioning

Participants highlighted that discharge coordination should be anticipated during hospitalisation with the outpatient healthcare professionals by discussing the patient’s care plan and sending the prescription and the discharge letter to all healthcare professionals before discharge. Specifically, community pharmacists and their teams should be included in coordination to ensure medication availability:

I think it’s a good idea to organise the discharge by transferring the information to the pharmacy and other parties 24 hours in advance, if possible. That way, we have time to contact the primary care physician or to order a specific medication. – Communitypharmacist, FG1

Participants suggested using checklists during hospitalisation and discharge to establish the teamwork process, standardise the discharge documents, secure information transfer, and increase interprofessional work. To ensure that patients understand their medications, participants suggested setting up quality standards for oral and written information to give to patients.

### Collaborative Leadership

#### Current State of Collaborative Leadership

Collaborative leadership was rarely mentioned, but participants discussed professional responsibility during the care transition. They discussed collective and individual responsibilities for patients and who should take responsibility during the transition period:

We [=hospital physicians] have a big responsibility in prescription changes at discharge, to make sure that the new treatment is well understood and followed [...] yet each professional is responsible and must do his job properly. – Hospital physician, FG2

#### Improving Collaborative Leadership

To enhance collaborative leadership, some participants suggested implementing an additional healthcare professional, such as a liaison HCP, who could oversee and ensure patient continuity of care. Others suggested defining a care coordinator from the hospital or the outpatient setting, such as the patient’s GP:

We should define that the patient is at the centre, that the general practitioner is the referent, and that he or she must coordinate the rest with the healthcare team. – Community pharmacist, FG4

### Interprofessional Conflict Resolution

No conflict resolution was discussed by participants in terms of the current state and areas for improvement.

### Macrolevel Improvements

At the macro level, participants stressed the need to enhance coordination among healthcare institutions and professional associations to clearly define roles, foster collaborative practices, and align on shared objectives. They highlighted that a more unified approach would reduce fragmentation within the system and lead to more consistent care delivery across regions:

Each HCP] has their little initiatives. We are not coordinated as one healthcare system, […] ultimately, we have the same patients, and we need to come together and say: “Here we are, we need to go ahead and implement this or that” – Community pharmacist, FG4

Additionally, participants underscored the necessity of political commitment to allocate resources effectively and provide structural support for interprofessional collaboration. This would involve financial investment and policy frameworks that promote integrated care models, enhanced communication channels, and ongoing interprofessional education programs.

## Discussion

This qualitative study involving patients and healthcare professionals provided valuable insights into interprofessional collaboration in medication management during the transition from hospital to outpatient care, using a serial focus group methodology that facilitated consensus-building among participants. The findings showed that while interprofessional communication and patient partnership are standard practices, there remains a significant lack of role clarification and collaborative leadership in the care transition. Participants are ready to collaborate and suggested several multi-level interventions to address these issues and emphasised the need for more robust policy support, including policy reforms and increased funding, to facilitate sustainable interprofessional collaboration and promote a culture of integrated care. Interestingly, interprofessional conflict resolution was not discussed, suggesting that the topic may be irrelevant to the participants at an early stage of collaborative practice.

Our findings align with previous studies that identified barriers to a seamless transition, including inadequate information transfer, limited availability of healthcare professionals, and unclear role definitions.^2^,^14^,^34^,^58^ Comparing our findings to the existing literature on interprofessional collaboration more broadly, several recommendations for improvement have already been suggested, such as clarifying roles, interprofessional training and enhanced policy support.^59–63^ Studies on care transitions have proposed practical improvements, some of which impact interprofessional collaboration, such as developing checklists, implementing electronic health records, and using technological tools to enhance communication.^2^,^8^,^34^,^64^ However, there is a significant gap in the literature: few studies have focused on the current and future state of interprofessional collaboration during care transition in both hospital and outpatient settings with front-line healthcare professionals.^29^ Geese et al similarly explored interprofessional collaboration and reported comparable findings, but their work focused primarily on hospital-to-home transitions for complex patients and did not include pharmacists as participants.^29^ Our study offers a unique contribution by using an established interprofessional framework, the CICH Framework.^32^ This approach allowed us to identify key issues and actionable improvements across the micro-, meso-, and macro-levels. Our findings (Figure 2) could serve as a valuable preliminary foundation for improving interprofessional collaboration during care transitions, offering a structured approach that could be adapted to various healthcare settings.

When comparing the current practice observed in our study to the continuum of interprofessional collaborative Framework by Careau et al, collaboration between hospital and outpatient care tends to be more multidisciplinary rather than truly interprofessional, focusing on the exchange of information rather than on the organisation of coordinated action with shared goals.^65^ The absence of effective coordination and clearly defined roles results in healthcare professionals communicating reactively by addressing issues only when questions or problems arise rather than proactively. This reactive approach leads to challenges in reaching the appropriate healthcare professional, potential delays, the risk of fostering defiance instead of trust, and a reduction in the overall quality of patient care. In the context of medication management, this type of reactive communication has been shown to lead to incomplete or delayed information transfer, increasing the likelihood of medication errors or lapses in patient care.^66^,^67^ A more proactive approach, where communication is initiated earlier and includes planning and coordination, could improve the continuity of care, ensure timely interventions, and enhance patient safety and interpersonal trust.^66^,^67^ Improving proactive communication through structured handovers and clear role definitions was rated as the most impactful by participants to anticipate potential issues, streamline processes, and promote collaborative leadership across care settings.

Role clarification is particularly challenging when team members are not geographically co-located and may have different goals and work cultures.^68^ This issue becomes especially critical during care transitions, where a clear leadership handover between settings is paramount but often unclearly defined.^7^ This leadership handover is often implied simply by sending the hospital discharge documents to the patient’s referring physician, which is sometimes encountered by patients weeks after discharge.^69^ This lack of clarity raises a pressing question: who should take the lead in ensuring continuity of care during this transitional phase, particularly in bridging the gap between hospital and outpatient services? The issue is further complicated in Switzerland, where the Diagnosis Related Group (DRG) billing system holds hospitals financially accountable for readmissions within 18 days for the same condition.^70^ To secure patient care and enhance care efficiency, it is essential to clearly define the roles of healthcare professionals during this transition by establishing clear leadership and communication pathways.

Our results reflect healthcare professionals’ strong interest in partnering with patients and family caregivers to improve care transitions. Patient education and empowerment are vital in care transitions, fostering self-management and medication adherence, both essential for long-term health outcomes.^2^,^71^,^72^ However, implementing effective patient partnerships should not be underestimated, as it requires the development of tailored educational materials, training healthcare professionals to engage patients effectively, and creating strategies that actively involve patients in their care.^73^,^74^

Participants in our study did not refer to formal guidelines in their discussions. Instead, they tended to rely on their usual practice or personal judgement to guide patient care, often adapting their approach to specific circumstances, suggesting variability in care delivery. In Switzerland, no clinical practice guidelines exist for securing hospital discharge. Swiss Patient Safety launched the “Medication Safety at the Interfaces” pilot project (2014–2017).^75^ It developed procedures for medication verification at hospital admission but focused only on transitions to inpatient care. Expanding its scope to include hospital discharge and outpatient care is now essential.

Despite growing interest in integrated care models at regional, cantonal, and national levels in Switzerland,^76^ our findings reveal a lack of clear guidance and policy support to define and promote interprofessional collaboration during care transition. Establishing a federation of healthcare associations could provide a foundation for interprofessional networks and education, allowing healthcare professionals to define roles, responsibilities, and shared goals together. Standardised protocols and guidelines could then implemented to improve information sharing, discharge planning, and medication reconciliation, facilitating the adoption of evidence-based practices and continuous quality improvement. A relevant example comes from the National Health Service in England, which has introduced guidance for hospital discharge and outpatient care.^77^ This guidance outlines healthcare professionals’ legal roles and responsibilities and provides recommended procedures for discharging patients and ensuring adequate community support.^77^ To achieve similar progress in Switzerland, policy support and political decisions from local and national authorities are necessary to provide clear guidance, establish common objectives, reassess the healthcare professionals’ remuneration system, and allocate additional resources to foster interprofessional collaboration across settings.^48^,^76^ Furthermore, the integration of interprofessional education at both pre-graduate and post-graduate levels ensures that healthcare professionals acquire the collaborative skills necessary to enhance teamwork, clarify roles, and improve communication to optimise collaboration in practices and patient outcomes.^63^

## Limitations

This study has several limitations. First, there may be a selection bias in recruiting participants, as participants may have had a greater interest in interprofessional collaboration than the broader population of healthcare professionals and patients. Second, our study included only 12 participants; however, our serial methodology facilitated in-depth discussions, allowing us to reach data saturation in this group. Both patient participants in this study were female, which may limit the diversity of perspectives represented. As a next step, replication of the research by a different group of participants would be valuable to confirm the external validity of our results. Third, our study was conducted in a single urban setting, enabling us to better understand our specific context, but further research in diverse geographical and institutional settings is necessary to validate our findings in other contexts.

## Conclusion

Our study highlighted current practices and areas for improvement in interprofessional collaboration during the transition from hospital to outpatient care, touching on five of the six competency domains outlined in the CIHC framework. While existing practices prioritise communication and patient involvement, challenges remain in achieving role clarification and fostering collaborative leadership. The areas identified for improvement, including stronger patient empowerment, more explicit role definitions for healthcare professionals, and policy reforms, underscore the need for comprehensive, multi-level strategies to bridge gaps between hospital and outpatient settings. Ensuring effective care transitions requires policy support and coordinated efforts among healthcare institutions to implement sustainable interventions.

## Data Availability

The deidentified data supporting this study’s findings are available on request from the corresponding author.
